# Expanding Upon Genomics in Rare Diseases: Epigenomic Insights

**DOI:** 10.3390/ijms26010135

**Published:** 2024-12-27

**Authors:** Jia W. Tan, Emily J. Blake, Joseph D. Farris, Eric W. Klee

**Affiliations:** Center for Individualized Medicine, Mayo Clinic, Rochester, MN 55905, USA; tan.jia@mayo.edu (J.W.T.); blake.emily@mayo.edu (E.J.B.); farris.joseph@mayo.edu (J.D.F.)

**Keywords:** DNA methylation, rare diseases, review, epioutliers, allele specific methylation, epivariation

## Abstract

DNA methylation is an essential epigenetic modification that plays a crucial role in regulating gene expression and maintaining genomic stability. With the advancement in sequencing technology, methylation studies have provided valuable insights into the diagnosis of rare diseases through the various identification of episignatures, epivariation, epioutliers, and allele-specific methylation. However, current methylation studies are not without limitations. This mini-review explores the current understanding of DNA methylation in rare diseases, highlighting the key mechanisms and diagnostic potential, and emphasizing the need for advanced methodologies and integrative approaches to enhance the understanding of disease progression and design more personable treatment for patients, given the nature of rare diseases.

## 1. Introduction

Rare diseases (RDs) are medical conditions affecting fewer than 200,000 individuals in the United States and less than 1 in 2000 in the European Union [1]. While individual RDs affect a limited number of the population, on aggregate, RDs encompass more than 10,000 unique disorders [2] and impact approximately 300 million people [3,4]. The majority of RDs are Mendelian genetic disorders [5,6] and comprehensive genetic testing, using exome sequencing and more recently, genome sequencing, is often used to identify causal variation. Sequencing technology advancements have also led to increased use of multi-omic approaches for variant identification, interpretation, and prioritization, which may include phenomics, transcriptomics, proteomics, metabolomics, and epigenomics [7]. While most rare diseases are associated with genetic factors, there is growing recognition of epigenetic involvement, particularly DNA methylation (DNAm), as a significant contributor to a subset of RDs. This is exemplified by Mendelian disorders of the epigenetic machinery (MDEM) [8], also referred to as chromatinopathies [9]. These insights highlight the complementary relationship between genetic and epigenetic factors, offering new avenues for understanding pathogenesis and potential therapeutic interventions [8].

**Figure 1:**
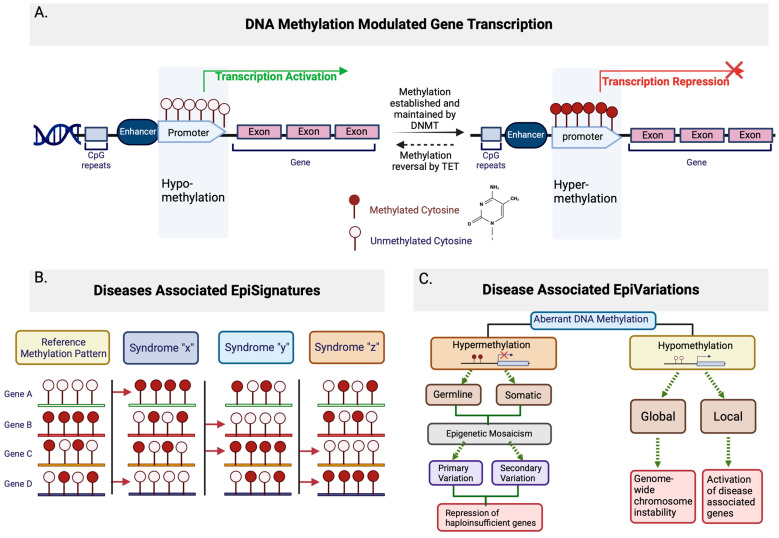
Schematic of DNA methylation modulated gene transcription and associated episignatures and epivariations. (**A**) General representation of the association between DNA methylation state and gene transcription. Hypomethyaltion in promoter generally leads to transcription activation, where the establishment and maintenance of DNA methylation through DNMT family proteins leads to hypermethylation in the promoter, which represses transcription. The methylation state can be reversed in a process initiated by the TET family proteins. (**B**) General representation of disease-associated episignatures. Left most represents the reference methylation patterns, with methylated or unmethylated CpGs within a gene. Hypothetical syndromes are shown with red arrows to indicate DNA methylation patterns at specific genes that differ from the reference methylation patterns. (**C**) General representation of disease-associated epivariations. Aberrant DNA methylation can be either hyper- or hypo-methylation. Hypermethylation can be either germline or somatic, primary or secondary, and ultimately leads to the repression of haploinsufficient genes. Hypomethylation occurs globally (genome-wide) or locally, global hypomethylation leads to chromosomal instability, while local hypomethylation leads to the activation of disease-associated genes.

DNA methylation is a crucial epigenetic modification involved in the regulation of gene expression. The most common and best-described methylation of DNA is the addition of a methyl group to carbon 5 of cytosine bases, forming 5-methylcytosine or 5mC [10] (Figure 1A). It is known that 5mC is often, but not exclusively, maintained in the context of cytosine-phosphate-guanine (CpG) dinucleotides [10]. DNA methylation patterns are commonly established and maintained by groups of DNA methyltransferases, such as the DNMT family and associated proteins [11]. The ten-eleven translocation (TET) family proteins mediate the reversal of 5mC methylation through iterative oxidation of 5mC to 5-hydroxymethylcytosine (5hmC), 5-formylcytosine (5fC), 5-carboxylcytosine (5caC) and a complete reversal is achieved by thymine-DNA glycosylase (TDG)-mediated abasic site (AP) and base excision repair (BER) [11]. It is believed that genetic variants that alter the functions of these regulatory proteins can lead to aberrant DNA methylation patterns, including hypermethylation (excessive methylation) or hypomethylation (reduced methylation), subsequently affecting gene expression patterns and chromatin structure [12]. In summary, DNA methylation is one of the primary epigenetic mechanisms that coordinates gene activity at the transcriptional level and regulates critical developmental and physiological pathways. As such, dysregulation of methylation is an important contributor to the manifestation of RDs [13,14].

## 2. DNA Methylation and Rare Diseases

DNA methylation profiles have been extensively investigated using DNA methylation microarrays to identify epigenetic dysregulation in many RDs [15]; reviewed in [16]. The resulting distinct and stable DNA methylation signatures that are induced by pathogenic variants in disease-causing genes, e.g., [5,12,17,18,19], coined “episignatures”, have been associated with more than 70 Mendelian conditions [7,20] (Figure 1B). The number of novel diagnostic episignatures is on the rise, with many identified for RDs, including α-thalassemia mental retardation syndrome (MIM: 300032) [21], Kabuki syndrome (MIM: 602113 and 300128) [22], CHARGE syndrome (MIM: 214800) [22], Sotos syndrome (MIM: 606681) [23], Floating Harbor syndrome (MIM: 611421) [24], Coffin–Siris syndrome, and other BAFopathies (MIM: 135900, 614607, 614608, 614609, and 615866) [25]. Episignatures have shown the capabilities to distinguish between different genetic conditions [26], support variant classification by defining phenotypic specificity, and are effective diagnostic modalities for rare Mendelian conditions following inconclusive testing [27,28]. As Sadikovic et al. (2021) reported, the validation rate for VUS reclassification in a selected cohort of patients with previous ambiguous/inconclusive genetic findings can reach 35% using methylation signature (EpiSign) analysis [28].

Episignatures are typically discovered using epigenome-wide association studies (EWAS) investigating DNA methylation patterns from microarrays, followed by the application of a multiclass machine learning classifier [29]. Moreover, the use of artificial intelligence tools for epigenomic studies in RDs has increased over the last decade [30]. For example, Aref-Eshgi et al. (2019) introduced a computational model using whole genome methylation data to aid in diagnosing 14 neurodevelopmental disorders characterized by known episignatures [24]. The model effectively identified methylation profiles suggestive of specific Mendelian conditions for 31% (21/67) of individuals with uncertain diagnosis, including cases where conventional molecular testing failed to identify any candidate variants. In addition, Turinsky et al. (2020) created EpigenCentral, a free portal to interactively classify and analyze epigenome data for known disease-associated episignatures [29]. Further optimization of episignature classifiers is important to improve model sensitivity and molecular diagnosis of Mendelian disorders of the epigenetic machinery. Walsh et al. (2024) introduced a machine learning method that uses an age- and sex-stratified methylation model for outlier detection, which significantly reduces false negatives in array-based methylation signature analysis. By accounting for age- and sex-related methylation changes, this approach improved the classification of samples with potential methylation-associated congenital disorders [31]. Oexle et al. (2023) trained episignature classifiers to robustly detect low-level mosaics while also revoking erroneous exome calls of mosaicism to highlight improved diagnostic yield for RDs [32].

In addition to episignatures, epivariations and epimutations, can also be detected by methylation microarrays. The term “epimutation” was originally intended to describe epigenetic changes that occur without alterations in the underlying DNA sequence [33]. Over time, it has been applied more broadly to various epigenetic changes [34]. The term “epivariations” was later introduced to describe rare epigenetic aberrations [30,35]. While the two terms are now often used interchangeably, “epivariation” generally refers to regions exhibiting aberrant methylation patterns, characterized by significant enrichment in epimutations, which are abnormal mutational changes that do not change the DNA sequence [35,36] (Figure 1C).

Epivariations, or in some literature, epimutations, can be subdivided into primary or secondary types based on their origin [37]. Primary epivariations are thought to arise from stochastic (random) errors in the establishment or maintenance of the epigenome by the DNA methyltransferase proteins family [38]. These errors are sporadic and not necessarily linked to the changes in the DNA sequences, such as certain types of imprinting anomalies seen in Prader–Willi (MIM: 176270) and Angelman Syndromes (MIM: 105830) [33]. On the other hand, secondary epivariations derive from underlying changes in local DNA sequence, including copy number variations (CNVs), where segments of DNA are duplicated or deleted, or single nucleotide variations (SNVs), which are single base changes in the DNA sequence [37] at differentially methylated loci. Additionally, mutations that disrupt regulatory elements [38] and expansions of CpG-rich tandem repeats (STR) [39]—repetitive sequences rich in CpG sites—are believed to contribute to changes in the local DNA sequence. For example, Fragile X syndrome (MIM: 309550) is caused by secondary epivariations in the FMR1 gene [40]. Both epivariation types are found in patients with RDs.

It is important to note that next-generation sequencing (NGS) technologies, including targeted (e.g., reduced representation bisulfite sequencing or RRBS), offer high specificity for targeted CpG-rich regions and sensitivity for detecting small changes in methylation levels at individual CpG sites. Whole genome (e.g., whole genome bisulfite sequencing or WGBS, enzymatic methyl sequencing or EMseq) provides comprehensive coverage for CpG sites across the entire genome, which allows higher sensitivity for detecting methylation changes even in non-CpG regions. Additionally, long-read sequencing (LRS) provides higher resolution of complex genomic regions and improves assembly quality, generating methylation profiling data that can help clarify variant pathogenicity and aid in the diagnosis of RDs [14]. Moreover, the usage of optical genome mapping (OGM) has enhanced the detection of large structural variants (SVs), CNVs, and repetitive sequence motifs at the single-cell level, enabling a more detailed characterization of cellular heterogeneity [41].

Several published studies have illustrated epigenomic approaches for improving the diagnosis of rare diseases and shortening diagnostic journeys for patients. Gatto et al. (2017) identified a rare pathogenic variant in *DNMT3B* by studying the methylation profiles in immunodeficiency-centromeric instability-facial anomalies syndrome 1 (ICF1; MIM: 242860) through RRBS [42]. Sun et al. (2014) studied genome-wide DNA methylation profiles of hereditary sensory and autonomic neuropathy type 1E (HSAN1E) patients with *DNMT1* mutations using WGBS and discovered all chromosomes hypomethylated with enrichment of NAD+/NADH pathway-associated genes in differentially methylated regions (DMRs) [43]. More recent examples include Smith et al. (2021) which found that loss-of-function variants of *DNMT3A* lead to decreased global DNA methylation in Tatton–Brown–Rahman syndrome (TBRS; MIM: 615879) [44]. Zhu et al. (2022) utilized WGBS to identify autism spectrum disorder (ASD)-associated methylation changes with an enrichment of DMRs in ASD-associated genes [45]. Miller et al. (2020) leveraged targeted long-read sequencing to identify the cause of altered *GNAS* exon A/B methylation in autosomal dominate pseudohypoparathyroidism type 1b (PHP1B; MIM: 603233) [46]. In summary, multiple studies have demonstrated the power of DNA methylation profiling as a valuable functional tool to aid in the diagnosis of RD cases, complementing standard genomic sequencing to unambiguously identify and interpret variation [47,48,49].

## 3. Limitations

The reported diagnostic yield among patients with neurodevelopmental disorders varies by method, with chromosomal microarray achieving a yield of 15–20% [50] and 30–40% for exome sequencing [51]. When compared to these benchmarks, DNA methylation profiling has demonstrated a diagnostic yield of approximately 30% in a selected cohort exhibiting features suggestive of rare neurodevelopmental conditions [28,52]. While promising, the described episignature and epivariation detection methods are not without limitations. With the number of identified episignatures and epivariations steadily growing, the specificity of the signature for different cell types and tissues remains to be defined. Epigenetic profiles vary between tissues and show cell-type heterogenicity which hamper EWAS studies that are primarily conducted using data derived from whole blood specimens [53,54]. For tissues that are not always easily accessible, such as the brain, using blood as a surrogate may not fully capture the tissue-specific methylation pattern due to variability in correlation, as observed in previous studies [55,56]. In addition, the lack of consensus methods to correct for population heterogeneity (e.g., genetic background, environmental exposures, and demographic factors) in disease cohort samples limits the reproducibility of analysis across methods and impacts subsequent result interpretation [57]. For example, genetic background can influence baseline methylation patterns, making it challenging to distinguish disease-associated changes from population-specific variations. Similarly, environmental exposures, such as smoking or diet, can alter methylation profiles independently of disease status. Demographic factors, like age and sex, are associated with dynamic and tissue-specific methylation changes. These challenges have been reported in studies [57,58] describing the influence of experimental design, training data size, normalization method, and effect size as limitations of episignature generation. It was observed that the lack of consensus methodology led to the generation of different episignatures for similar pathologies [17,23,59]. Moreover, although improvements have been made to enhance the detection of genetic mosaicism [32,60], existing DNA methylation array technologies remain susceptible to molecular misdetection and fail to detect low-level mosaicism, with the reported resolution limit for detecting mosaicism being 10–15% [60]. There is also a reliance on pre-existing characterized episignatures to identify Mendelian disorders, together reducing diagnostic sensitivity [61]. Additionally, the expansion on syndrome-specific episignatures should be considered, as exemplified by the paralogous genes *CREBBP* and *EP300*. Loss-of-function variants in either gene cause Rubinstein–Taybi syndrome (RSTS), whereas pathogenic gain-of-function missense and in-frame indel variants in exons 30 and 31 lead to Menke–Hennekam syndrome (MKHK). While the current MKHK subtype categorizations are gene-specific (subtype 1 for *CREBBP*; subtype 2 for *EP300*), the observed distinct domain-specific episignatures in MKHK subtypes suggest the need for a more nuanced, domain-specific categorization [62,63]. The clinical interpretation of rare epivariants can also be challenging, especially within intragenic regions or genes not yet associated with the patient’s phenotype [64].

## 4. Methylation for Single Patient (N = 1) Rare Disease Studies

In addition to the limitations discussed above, conventional group vs. group or multigroup comparisons with appropriate cohort sizes to meet statistical significance in standard randomized control trials are often not feasible for studying RDs [65,66]. Rare diseases often have very few reported patients, resulting in small and heterogeneous cohorts where canonical group comparison method assumptions are not met, particularly in DNA methylation studies that utilize bisulfite sequencing. These group comparison methods, such as t-tests or ANOVA, assume independence of observations, normality of data, homogeneity of variances, and sufficient sample size to detect statistically meaningful differences in methylation patterns [67]. Despite ongoing sequencing efforts, the lack of publicly available population epigenome datasets as a benchmark to study changes in DNA methylation creates analytical challenges [64]. In addition, without adequate control groups, the ability to capture interpatient heterogeneity, including disease presentation, progression, genetic makeup, and environmental exposures, is often complicated. Within the context of rare diseases, similarities in aberrant CpG methylation patterns have been observed in certain neurodegenerative diseases when targeting a defined gene set [68]. However, disease-associated aberrant DNA methylation at a specific locus in an individual is presumably significantly different from individuals with unrelated phenotypes or control group cohorts [69]. Outlier strategies for identifying potentially causal findings in transcriptomic studies have improved rare disease diagnosis and discovery [70,71]. More recently, investigating methylation levels to understand outlier effects has facilitated the diagnosis of unsolved rare disease cases [31,72,73].

**Figure 2:**
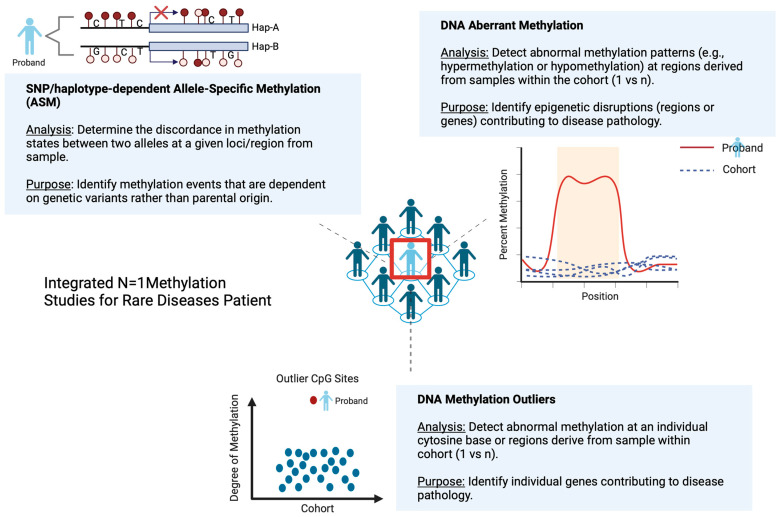
Schematic representation of integrated N = 1 methylation studies for rare disease patients. Possible integrated approaches include SNP/haplotype-dependent Allele-Specific Methylation (ASM), aberrant DNA methylation, and DNA methylation outlier studies. The purpose of the integration is to capture the epigenetic landscape of the proband within a rare disease cohort, which could provide useful insight into rare disease pathology, leading to improved diagnosis and management.

Cheung et al. (2023) proposed that screening for outlier methylation events genome-wide could aid in the identification of coding and noncoding, functional rare SNVs and structural variants (SVs) in unsolved rare cases to improve the diagnosis rate [74]. The authors further demonstrated that rare methylation outliers are heritable and proximally linked to causal rare noncoding or complex SV events using long-read sequencing. Further, Oliver et al. (2021) presented *BOREALIS*, a tool to identify outlier methylation events using sequence-based methylation data as a novel avenue of exploration in undiagnosed cases of rare disease [66]. These studies highlight the potential of methylation outlier detection methods as a complementary approach to increase diagnostic rates in rare disease patients (Figure 2).

Allele-specific methylation (ASM) is another phenomenon that may contribute to rare disease diagnosis. ASM events are reportedly increased in cancers, including lymphoma and myeloma, due to global allele-specific CpG hypomethylation [75]. ASM is considered a hallmark of both genomic imprinting, where the methylation of an allele is determined by its parent-of-origin, and non-imprinted status, or haplotype-dependent ASM (hap-ASM).

Hap-ASM refers to the difference in methylation state between two alleles in a heterozygous individual. This phenomenon occurs when *cis*-acting polymorphisms, such as single-nucleotide polymorphisms (SNPs), influence the methylation status of nearby CpGs. These SNPs can alter transcription factor (TF) binding, which affects the recruitment of methyltransferases and subsequently modulates the methylation landscape of one allele compared to the other. In hap-ASM regions, the presence of heterozygous SNPs is commonly observed, they are responsible for genetic differences between the two homologous chromosomes [76,77,78].

Traditionally, hap-ASM was assessed directly by WGBS or methylation quantitative trait loci (mQTL) analysis, which correlates the net methylation of single CpGs with genotypes at nearby SNPs [79]. For these methods, simultaneous observation of the DNA methylation state within each genetic variant of a haplotype (i.e., allele) and the allele of origin are needed [80]. The lack of ASM detection tools has historically limited research into its potential as a diagnostic method; however, the recent development of several ASM detection tools—MethPipe [81,82], MONOD2 [83], MethHaplo [84], DAMEfinder [85], and CpelAsm [86]—have expanded the possibilities for genome-wide ASM analysis. These advancements have made it possible to identify ASM events, potentially opening the door for the using ASM in the diagnosis of complex cases in RD patients.

Identification of methylation outliers or ASM events alone may not be sufficient to assess the causal relationship between a variant and an RD phenotype. Understanding the allelic specificity of these methylation events is critical in attributing observation to a suspected RD diagnosis. Cheung et al. (2023) reported that 80% of rare hypermethylation events from an RD cohort appeared to be allele-specific [74]. Non-diagnostic exome sequencing is typically followed by genome sequencing and transcriptome profiling, where expression outlier identification is often coupled with allele-specific expression (ASE) and structural variant analysis to determine allelic specificity and identify pathogenic SVs [64]. As methylation studies are increasingly used for RD diagnosis, similar strategies using correlated evidence should be pursued (Figure 2).

The limited number of tools available to study methylation in the scope of RDs and the dependency on accurately phased data to distinguish maternal and paternal alleles impose significant challenges. Most current phasing methods do not use parental data [87]; instead, the calculation of alleles from each chromosome, or subchromosomal phase block, are grouped into two haplotypes. Subsequent ASM events are then inferred from the identified haplotypes without a clear understanding of the allele or parent of origin. This creates a burden in terms of analysis and result interpretation when sequence data from at least one parent are not available. The emerging use of long-read sequencing has begun to overcome this issue, as tools such as MethPhaser [88] and PatMat [87], improve the quality of phasing through parental data inclusion. Epigenomics has the potential to significantly expand and improve clinical testing modalities in rare disease patients; however, the development of guidelines and standardized workflows is critical in a rapidly growing field.

## 5. Future Perspectives

There is increasing global awareness and attention to the impact of rare diseases on human health, with studies demonstrating the importance of early and unambiguous diagnosis. Despite significant advancements in sequence-based testing over the last decade, most patients suspected of having a rare genetic disease remain undiagnosed. As answers are sought for undiagnosed patients, studies are evaluating the intricate interplay between epigenetic mechanisms and disease pathogenesis. Methylation studies may provide valuable data to complement traditional genomic testing and significantly advance diagnostic and therapeutic strategies. Such studies will benefit from increased adoption of long-read technologies, where methylation signals can be obtained directly from the genomic sequencing of a sample. However, before DNA methylation profiling can be broadly implemented, several gaps require further attention and exploration. The presence of comprehensive, open reference databases containing epigenomes of individuals across different tissues and cell types is needed. Loyfer et al. (2023) highlighted the importance of addressing this deficiency, emphasizing the need for robust reference datasets that accurately capture the epigenomic landscape of healthy individuals [89]. Methylation reference datasets serve as invaluable resources for researchers seeking to compare epigenetic patterns observed in individuals affected by rare diseases with those in healthy populations. The availability of large-scale, well-annotated reference datasets will enable broader accessibility, establish a comprehensive benchmark, and foster interdisciplinary collaboration to train complex algorithms to improve clinical utility and diagnostics.

It is also critical to collaboratively invest in developing standardized analytical pipelines for epigenomic data to ensure the reproducibility and reliability of findings derived from individual rare diseases. Harmonized data generation, processing, and interpretation across research laboratories will facilitate cross-study comparisons and meta-analyses. DNA methylation analysis has the utility to become another critical care tool for RD patients. Early diagnosis using epigenome profiling may support the implementation of interventions through behavioral therapy, specialized learning programs, and individualized medicine [16].

In conclusion, technological advancements have not only significantly advanced our understanding of the role of epigenetics in rare diseases, but they have also provided valuable insights into the diagnosis of rare diseases through the various identification of episignatures, epivariation, epioutliers, and ASMs. Identifying the links between these epigenetic factors and the underlying disease phenotype requires robust basic research efforts. Moreover, overcoming the limitations outlined depends on sustained funding for current and future rare disease research programs, as the associated costs can accumulate for both the researchers and patients.

Looking forward, that with the future establishment of comprehensive reference databases, promotion of standardization efforts, and encouragement of data sharing and collaboration will unlock the full potential of epigenomic studies to support the diagnosis and management of rare diseases. These developments will improve diagnostic precision and support the creation of more effective, personalized (targeted) therapies for individuals affected by rare diseases.
